# Diagnostic accuracy of multiplex polymerase chain reaction on tissue biopsies in periprosthetic joint infections

**DOI:** 10.1038/s41598-021-99076-4

**Published:** 2021-09-30

**Authors:** Igor Lazic, Susanne Feihl, Peter M. Prodinger, Ingo J. Banke, Andrej Trampuz, Rüdiger von Eisenhart-Rothe, Christian Suren

**Affiliations:** 1 Department of Orthopaedic Surgery, School of Medicine, Klinikum rechts der Isar, Klinik für Orthopädie und Sportorthopädie, Technische Universität München, Ismaninger Str. 22, 81675 Munich, Germany; 2 Department of Orthopaedic Surgery, Krankenhaus Agatharied, Hausham, Germany; 3 Centrum für Muskuloskeletale Chirurgie, Charité—Universitätsmedizin Berlin, Berlin, Germany

**Keywords:** Microbiology, Pathogens, Medical research, Translational research, Molecular medicine

## Abstract

The diagnosis and treatment of periprosthetic joint infection (PJI) currently relies on cultures, which are time-consuming and often fail. Multiplex PCR assays promise reliable and prompt results, but have been heterogeneously evaluated. In this study, we analyse multiplex PCR in pathogen identification using only tissue biopsies. 42 patients after revision arthroplasty of the hip or knee were evaluated using multiplex PCR to identify microorganisms. The patients were classified according to the diagnostic criteria published by Zimmerli et al. and the results were compared to the respective microbiological cultures. PJI was detected in 15 patients and 27 revisions were aseptic. The multiplex PCR of tissue biopsies had a sensitivity of 0.3 (95% CI 0.12–0.62), a specificity of 1.0 (0.87–1.0), a positive predictive value of 1.0 (0.48–1.0) and a negative predictive value of 0.73 (0.56–0.86). The diagnostic accuracy of multiplex PCR on tissue biopsy samples is low in comparison to routine microbiological cultures. The evaluation of tissue biopsies using multiplex PCR was prone to false negative results. However, multiplex PCR assays have the advantage of rapid pathogen identification. We therefore recommend further investigation of multiplex PCR in the setting of suspected PJI with a careful choice of specimens.

## Introduction

The diagnosis of periprosthetic infection (PJI) represents a great challenge in clinical practice. The distinction between infected and non-infected arthroplasties has considerable implications on the further treatment and outcome^1^. Currently, the established diagnostic algorithms for PJI demand the consideration of pre- and postoperative parameters, which combined lead to a sufficient diagnostic accuracy overall^1,2^. However, preoperative pathogen identification is not mandatory to diagnose PJI. In clinical practice, information about the underlying pathogen might not be available at the time of surgery^1^. The identification of the pathogen still relies on microbiological cultures, which can have incubation times of several days in the case of slow-growing organisms typical for low-grade PJI. Furthermore, the identification of the underlying pathogen in conventional microbiological cultures of preoperative joint fluid aspirate often fails^1,3,4^.

The importance of accurate organism identification and susceptibility testing prior to surgery to avoid a delay of pathogen-specific therapy has been shown^5–7^. Molecular diagnostic methods such as polymerase chain reaction (PCR) have already been evaluated in the setting of PJI and offer the advantage of pathogen identification within hours^8–10^. However, commercially available multiplex PCR assays with panels of various potential pathogens have demonstrated heterogeneous results^11–17^. Most studies concerning multiplex PCR assays have evaluated samples of joint or sonication fluid^24–29^. However, in some cases, preoperative joint aspiration is problematic, and some latency remains due to processing before sonication fluid for PCR analysis is available. Furthermore, in some cases, the suspicion of PJI arises within the procedure, when a collection of synovial fluid is no longer feasible. In such cases, analysis of tissue samples with a PCR method might still yield timely results. Therefore, in this study, we assessed the value of multiplex PCR with an expanded panel in terms of microbial gene identification using only intraoperative tissue biopsies.

## Patients and methods

Synovial fluid and tissue from a prospective cohort of patients who underwent elective revision surgery of total hip or knee arthroplasty at our institution in 2009 were enrolled. No antimicrobial therapy was administered for at least 2 weeks before surgery. The study was approved by the ethics commission of the Technical University of Munich (Ethikkommission der Technischen Universität München) under reference no. 2544/09. The study was conducted in accordance with the regulations of the ethics commission. A written informed consent was obtained from all the patients participating in the study. The patients were classified into the periprosthetic infection or aseptic revision group according to the diagnostic criteria published by Zimmerli et al., which were then in use at our institution^6^. Thus, periprosthetic infection was diagnosed in cases with a present sinus tract, purulence in the joint, or a pathogen identified in at least two microbiological cultures of synovial fluid or intraoperative tissue biopsies. In addition, the following parameters were evaluated: synovial leukocyte count, elevated serum C-reactive protein (CRP), blood leukocyte count, single-positive microbiological cultures, ESR and histology classified according to the criteria published by Morawietz and Krenn^18^. A combination of histology indicating infection and another positive parameter led to the diagnosis of PJI, as well.

Five biopsies from the periprosthetic tissue were analyzed per patient using microbiological cultures. One tissue biopsy per patient was frozen and stored at − 80 °C. Automated multiplex PCR analysis was performed on thawed samples using the Unyvero A50 Analyzer (Curetis AG, Holzgerlingen, Germany) per the manufacturer’s instructions to identify the organism and respective resistance genes. Detection of an organism was ruled positive if the specimen exceeded 104 DNA fragments/pathogen/ml.

For microbiological analysis, synovial fluid and tissue biopsies were separately cultured on aerobic and anaerobic plates (Columbia sheep blood agar, Columbia chocolate agar, McConkey agar, Schädler anaerobic agar and Schädler KV anaerobic agar), as well as in liquid media (thioglycolate and glucose broth) at 37 °C. After 24 h, 48 h and 10 days, bacterial growth was evaluated. Microbial identification and antibiotic susceptibility testing were performed using Vitek2 (bioMerieux, Nürtingen, Germany). Results of the PCR analysis were available within 6 h. Preliminary results of the microbiological cultures of tissue samples were available 24 h after plating at the earliest. Large discrepancies of turnaround times result from these different methodological approaches and were therefore not indicated.

Statistical quality criteria were calculated and compared using a binary classification test. Sensitivities, specificities, positive and negative predictive values, and positive and negative likelihood ratios were estimated and assessed with Fisher's exact test. To compare the different methods, the overall accuracy and Fleiss’s extension of Cohen's kappa were calculated. For categorical data, absolute frequencies are presented. For age, the mean and standard deviation are given. All analyses were performed using the statistical software IBM SPSS Statistics (Version 22.0. Armonk, NY: IBM Corp.).

## Results

Forty-two patients (19 men and 23 women) with a mean age of 70.0 ± 12.6 years were included. Revision surgery of 24 total hip arthroplasty and 18 total knee arthroplasty procedures was performed. PJI was detected in 15 patients, and 27 revisions were aseptic. Aseptic revision cases consisted of 14 cases of aseptic loosening in THA, nine cases of aseptic loosening in TKA, one case of impingement in THA, one case of malalignment, one case of material failure and one case of patella baja and arthrofibrosis in TKA.

The microbiological cultures of joint fluid showed growth in 66.6% (10/15) of patients classified as septic but only in 3.8% (1/27) of aseptic revisions. Hence, a sensitivity of 0.67 (95% CI 0.38–0.88) and a specificity of 0.96 (0.81–0.99), with a positive predictive value (PPV) of 0.91 (0.59–1.00), a negative predictive value (NPV) of 0.83 (0.66–0.95), and a positive/negative likelihood ratio (LR) of 18.0/0.35, were demonstrated. The overall accuracy was 0.86. *Staphylococcus aureus* was detected preoperatively in one case of aseptic revision, which was not confirmed in a synopsis of the postoperative microbiological and histopathological results. All five preoperative culture-negative PJIs were later positive in intraoperative tissue biopsy cultures. In one case, a polymicrobial infection with *Dermatobacter hominis*, *S. aureus* and *Corynebacterium jeikeium* was detected, while the remaining four cases showed growth of coagulase-negative staphylococci. One case showed growth of *Streptococcus agalactiae* in synovial fluid culture, which could not be reproduced in tissue biopsy culture. Overall, in the tissue biopsy cultures, a sensitivity of 0.93 (0.68–1.00) and a specificity of 0.96 (0.81–1.00) with a PPV of 0.93 (0.68–1.00), NPV of 0.96 (0.81–1.00), and positive/negative LR of 25.2/0.07, were demonstrated. The overall accuracy was 0.95.

By multiplex PCR of tissue biopsies, 33.3% (5/15) of PJIs were correctly identified, while no pathogen was detected in the aseptic revisions. In one case, a mismatch was noted: multiplex PCR detected the presence of *Cutibacterium acnes*, although microbiological cultures showed growth of coagulase-negative staphylococci.

In one case of PJI caused by *Listeria monocytogenes*, verified in both synovial fluid and biopsy cultures, multiplex PCR detected only the presence of bacterial genetic material. However, the Unyvero second-generation cartridge does not contain primers for Listeria. Furthermore, in one case of polymicrobial infection, only *S. aureus* was detected, while *Dermabacter hominis* and *C. jeikeium*, which grew in biopsy culture, were not found by PCR. The pathogen identification by conventional cultures and PCR of tissue samples is specified in detail in Table 1. Therefore, multiplex PCR had a sensitivity of 0.3 (0.12–0.62) and a specificity of 1.0 (0.87–1.0), with a PPV of 1.0 (0.48–1.0), NPV of 0.73 (0.56–0.86) and positive/negative LR of 1.6/0.67. Its overall accuracy was 0.76. As a measure of the agreement of the three diagnostic methods, Fleiss’s kappa was estimated at 0.53 (Table 3). The results of the three diagnostic methods for each PJI are demonstrated in Table 2.

**Table 1:** Confusion matrix of pathogen detection by microbiological cultures and PCR of tissue samples.

	Tissue sample culture	
	Positive	Negative
**Tissue sample PCR**		
Positive	CNS (3), *Staphylococcus aureus* (1), *Streptococcus agalactiae* (1)	*Cutibacterium acnes* (2)
Negative	CNS (7), *Dermabacter hominis* (1), *Corynebacterium jeikeium* (1), *Corynebacterium amycolatum* (1), *Streptococcus salivarius* (1), Listeria monocytogenes* (1)	–

Number of patients with microbiological growth is indicated in brackets.

*No primers were included in the PCR test kit for Listeria.

**Table 3:** Overview of the microorganisms detected in PJI by microbiological cultures of joint aspirate and tissue samples and by PCR of tissue samples.

PJI no.	Joint aspirate culture	Tissue sample culture	Tissue sample PCR
1	CNS (*Staphylococcus epidermidis*)	CNS (*Staphylococcus epidermidis et hominis*)	–
2	CNS	CNS	CNS
3	CNS	CNS	–
4	CNS (*Staphylococcus epidermidis*)	CNS (*Staphylococcus epidermidis*)	CNS
5	–	CNS	*C. acnes*
6	CNS (*Staphylococcus epidermidis*)	CNS (2 biotypes *Staphylococcus epidermidis*)	–
7	–	*Dermabacter hominis* *S. aureus* *Corynebacterium jeikeium*	*S. aureus*
8	–	CNS (*Staphylococcus epidermidis*)	CNS *C. acnes*
9	Listeria monocytogenes*	Listeria monocytogenes*	Bacteria detected
10	–	CNS	–
11	–	CNS	–
12	*Corynebacterium amycolatum*	*Corynebacterium amycolatum* CNS	–
13	*Streptococcus salivarius*	*Streptococcus salivarius*	–
14	*S. agalactiae*	–	–
15	*S. agalactiae*	*S. agalactiae*	*S. agalactiae*

CNS = coagulase-negative staphylococci.

**Table 2:** Evaluation of the different diagnostic tools applied, n = 42.

Criteria	Joint aspirate culture	Tissue sample culture	Tissue sample PCR
*P* value	< 0.0001	< 0.0001	0.0035
Sensivity [95% CI]	0.67 [0.38, 0.88]	0.93 [0.68, 1.00]	0.3 [0.12, 0.62]
Specificity [95% CI]	0.96 [0.81, 0.99]	0.96 [0.81, 1.00]	1.0 [0.87, 1.0]
Positive predictive value [95% CI]	0.91 (0.59, 1.00]	0.93 [0.68, 1.00]	1.0 [0.48, 1.0]
Negative predictive value [95% CI]	0.83 [0.66, 0.95]	0.96 [0.81, 1.00]	0.73 [0.56, 0.86]
Likelihood ratio	18.0	25.20	–
Overall accuracy	0.86	0.95	0.76
Fleiss' kappa	0.53 (moderate)		

Per patient, five to six tissue specimens were taken for culture, and one specimen was taken for tissue PCR.

## Discussion

Microbiological cultures are the gold standard approach to identify the underlying pathogen in PJI. However, the sensitivity of cultures is merely satisfactory: the sensitivity of synovial fluid cultures ranges between 45 and 75%^19,20^ and of tissue sample cultures between 65 and 94%^21–23^, respectively. In this context, rates of culture-negative PJI infections between 7 and 22% have been reported^24,25^. The treatment of culture-negative PJIs is more challenging, and their outcomes are more heterogeneous^25,26^. A recent study demonstrated unacceptable rates of treatment failure of up to 53.1%^25^. Given such poor outcomes associated with culture-negative PJI, prompt identification of the underlying organism is paramount and has to be achieved by any means necessary. Recent evidence suggests that molecular diagnostic methods may provide increased sensitivity^14,15,25^. Several studies applying the Unyvero ITI first-generation cartridge showed comparable results to conventional cultures when joint fluid aspirate and sonication fluid were analysed^11,12,16,27^. Malandain et al. conducted a retrospective multicentre study with 251 patients, comparing multiplex PCR using a first-generation cartridge with conventional culture and 16S rRNA PCR. The authors reported a low concordance rate of 39.8% between multiplex PCR and the gold standard^16^. Interestingly, Malandain et al. randomly selected the different sample types for multiplex PCR to balance their distribution (tissue samples and synovial fluid)^16^. Assuming we would be able to achieve a higher diagnostic accuracy by evaluating single sample types, we assessed the diagnostic accuracy of the Unyvero ITI second-generation cartridge using only tissue samples.

The agreement of the results achieved in this study using synovial fluid cultures, tissue sample cultures and tissue sample multiplex PCR would be statistically described as “moderate” (Kappa = 0.53). In our patient population, the sensitivity and specificity of conventional cultures were comparable to the results in the available literature^1^. However, we noted a severe discrepancy in the sensitivity between conventional cultures and multiplex PCR, which has already been described as a major limitation of multiplex PCR in PJI diagnosis^12,14,17^.

The first generation of multiplex PCR showed low sensitivity rates in general. Renz et al. evaluated the sonication fluids of 111 patients and found a sensitivity of 51%^17^. Hischbeth et al. obtained a sensitivity of 67% from an analysis of sonication and synovial fluids of 62 patients^14^. Lausmann et al. described a sensitivity of 79% using synovial fluid from 60 patients^12^. Recently, we evaluated the second-generation Unyvero ITI cartridge on joint aspirate specimens and demonstrated an improved diagnostic accuracy in comparison to routine cultures, with a sensitivity of 80%^10^. Similarly, Lausmann et al. demonstrated an increased performance of the novel Unyvero ITI cartridge when analysing synovial fluid of 97 patients, with a sensitivity of 85%^28^.

Although the Unyvero ITI second-generation cartridge was used in this study, the multiplex PCR of tissue samples yielded a sensitivity of only 30%, which is not acceptable for clinical practice. In general, the identification of pathogens by molecular diagnostics should be feasible at lower bacterial counts than those required for microbiological cultivation. Accordingly, Morgenstern et al. demonstrated that multiplex PCR was superior for the detection of low-virulence organisms, such as coagulase-negative staphylococci and *C. acnes*^11^.

A potential benefit of multiplex PCR is the simultaneous identification of several pathogens in polymicrobial infections that may compete during conventional cultivation and therefore be missed. However, out of the four identified cases of polymicrobial infections, one pathogen was detected in one of the cases by multiplex PCR, whereas the other three cases yielded negative PCR results. Interestingly, Bémer et al. demonstrated similar results in a prospective study of 299 specimens. The authors noted that 66% of polymicrobial infections tested positive for only one pathogen, and in 25% of polymicrobial infections, no pathogen was found by multiplex PCR^29^.

There are various possible causes that could explain the low accuracy of multiplex PCR of tissue biopsies in comparison to synovial fluid. First, only one frozen biopsy was available per patient, so that a sampling error might have influenced the amount of bacterial material within the specimens. These single tissue biopsies may not have been representative. Similarly, the inoculum of potential low-grade bacteria may be too low to allow for microbiological cultivation. To increase the probability of bacterial growth in at least one sample, three to five cultures are recommended in PJI diagnostics^22,30^. However, it must be emphasized that two different methodological approaches are being compared. PCR is a highly sensitive molecular method that should allow the detection of any bacterial material even in small samples and is not dependent on specific growth conditions. While the low sensitivity of the PCR in this study may be explained by a low amount of bacterial DNA in the single tissue samples, it would not necessarily be compensated by using more samples. Mandalain et al. analysed two tissue samples per patient and achieved a concordance rate of 58% between microbiological culture and multiplex PCR, which is comparable to our moderate results. Borde et al. reported a higher concordance rate of 82% when analysing three tissue samples using the Unyvero ITI first-generation cartridge. However, the authors state that this concordance was mostly due to matching negative results and that no superiority of multiplex PCR over conventional cultures should be postulated^31^.

Second, freezing and thawing could have reduced the sample quality. Hence, the remaining bacterial DNA might have been beneath the detection threshold, resulting in false negative classification^32^. However, we previously evaluated the Unyvero ITI second-generation cartridge on joint aspirate specimens, which were analysed in the same manner as presented here. We saw no detrimental effects caused by freezing and thawing of the samples^10^.

Third, the processing of the tissue samples, even though in accordance with the manufacturer’s protocol, may not have been adequate. Bémer et al. highlighted the risk of PCR inhibition caused by excessively high DNA concentrations and recommended testing diluted and undiluted DNA when performing multiplex PCR assays^29^.

The specificity of the multiplex PCR of tissue biopsies was exceptionally high and is in accordance with the literature^10,12,17,29^. We identified only one false positive case with *S. aureus* by conventional cultures in the aseptic cases, which has not been confirmed in the PCR. Although the thorough review of all diagnostic criteria made a periprosthetic infection with a highly virulent pathogen most unlikely, the detection of *S. aureus* led to an antibiotic treatment in clinical practice. However, retrospectively, this pathogen detection has to be considered as a contamination. However, this multiplex PCR assay has only a limited panel of organisms represented in the cartridges, and neglecting unrepresented bacteria is a potential risk. For example, one specimen showed cultivation of *L. monocytogenes*, which is not included in the panel, and returned only the presence of bacterial genetic material in the multiplex PCR.

There are several limitations of this study. First, the diagnostic algorithm for PJI used in this study was appropriate at the time of surgery but is outdated now. Currently, several more recently developed diagnostic algorithms are recommended, though no definitive gold standard exists^1^. All consist of various parameters, which have been subject to change over time^1^. Still, applying a more recent diagnostic algorithm as the gold standard might have changed our results. We must emphasize that every diagnostic algorithm at hand relies on an integrated view of long-established and more recent criteria, so that any single novel diagnostic method is difficult to judge by itself. Furthermore, we want to stress that a potential advantage of multiplex PCR is to identify the pathogen in a timely manner rather than to improve already well-established diagnostic algorithms.

It is certainly desirable to evaluate the worth of multiplex PCR of tissue biopsies in larger prospective trials including a higher number of patients with more than one sample each. The considerable cost of analysing each biopsy by PCR could be circumvented by pooling biopsies. Recently, we showed much more promising results using joint aspirate samples^10^. After this study, however, we conclude that in cases where multiplex PCR of synovial fluid is not feasible, the analysis of single tissue biopsies should be interpreted with caution. Furthermore, multiplex PCR assays are capable of identifying resistance genes, but no resistance genes were found in this study using molecular diagnostics even though conventional cultures revealed resistance. The possibilities for resistance gene identification using multiplex PCR are still limited, and the panels include only a few resistance genes. Sigmund et al. reported a sensitivity for the detection of resistance genes using multiplex PCR on sonication fluid of 51–71%. Similar to our findings, the authors reported a low detection rate of oxacillin and macrolide resistances, although the resistance marker genes (mecA, mecC) and (ermA, ermC) were included in the panel^33^.

In summary, the diagnostic accuracy of the multiplex PCR applied in this study using tissue biopsy samples is low in comparison to routine cultures. This approach poses a high risk of false-negative results. However, molecular methods such as multiplex PCR are easy to use and have the advantage of rapid pathogen identification. We therefore recommend further investigation of multiplex PCR in the setting of PJI, but the specimens should be selected with caution.

## Data Availability

Data are available from the authors upon reasonable request and with explicit permission of the participants.
